# Dual Agency in Hospitals: What Strategies Do Managers and Physicians Apply to Reconcile Dilemmas Between Clinical and Economic Considerations?

**DOI:** 10.34172/ijhpm.2021.87

**Published:** 2021-08-15

**Authors:** Ruth Waitzberg, Nora Gottlieb, Wilm Quentin, Reinhard Busse, Dan Greenberg

**Affiliations:** ^1^The Smokler Center for Health Policy Research, Myers-JDC-Brookdale Institute, Jerusalem, Israel.; ^2^Department of Health Policy and Management, School of Public Health, Faculty of Health Sciences, Ben-Gurion University of the Negev, Beer-Sheva, Israel.; ^3^Department of Health Care Management, Faculty of Economics & Management, Technical University Berlin, Berlin, Germany.; ^4^Department of Population Medicine and Health Services Research, School of Public Health, Bielefeld University, Bielefeld, Germany.; ^5^European Observatory on Health Systems and Policies, Brussels, Belgium.

**Keywords:** Dual Agency, Dilemma, Reconciliation Strategies, Israel, Germany, Activity-Based Payments

## Abstract

**Background:** Hospital professionals are "dual agents" who may face dilemmas between their commitment to patients’ clinical needs and hospitals’ financial sustainability. This study examines whether and how hospital professionals balance or reconcile clinical and economic considerations in their decision-making in two countries with activity-based payment systems.

**Methods:** We conducted 46 semi-structured interviews with hospital managers, chief physicians and practicing physicians in five German and five Israeli hospitals in 2018/2019. We used thematic analysis to identify common topics and patterns of meaning.

**Results:** Hospital professionals report many situations in which activity-based payment incentivizes proper treatment, and clinical and economic considerations are aligned. This is the case when efficiency can be improved, eg, by curbing unnecessary expenditures or specializing in certain procedures. When considerations are misaligned, hospital professionals have developed a range of strategies that may contribute to balancing competing considerations. These include ‘reshaping management,’ such as better planning of the entire course of treatment and improvement of the coding; and ‘reframing decision-making,’ which involves working with averages and developing tool-kits for decision-making.

**Conclusion:** Misalignment of economic and clinical considerations does not necessarily have negative implications, if professionals manage to balance and reconcile them. Context is important in determining if considerations can be reconciled or not. Reconciling strategies are fragile and can be easily disrupted depending on context. Creating tool-kits for better decision-making, planning the treatment course in advance, working with averages, and having interdisciplinary teams to think together about ways to improve efficiency can help mitigate dilemmas of hospital professionals.

## Background

**Key Messages**

** Implications for policy makers**

- Hospital professionals are “dual-agents” who may face dilemmas between their commitment to patients’ clinical needs and hospitals’ financial sustainability.
- In some situations, economic and clinical considerations are aligned, and activity-based payments promote efficiency. In case of misalignment of economic and clinical considerations, professionals sometimes manage to balance and reconcile them and thus mitigate negative impacts.
- In order to reduce health professionals’ dilemmas and enable them to balance different considerations, it is important to provide tools for decision-making such as clear instructions for treatment and for the coding of activities to maximize payments while avoiding undue manipulations. Other strategies used by hospital professionals to mitigate these dilemmas are interdisciplinary planning and decision-making and better planning of the treatment course in advance.
- Healthcare providers should be aware that some reconciliation strategies are fragile and can be easily disrupted, leading to unintended consequences such as negative selection of patients, under- or over-treatment, or poorer quality of care.
- The delicate balance between economic and clinical considerations depends on context, eg, the hospital market structure, the resources available in different countries, and professionals’ backgrounds and preferences.

** Implications for the public**

Hospital managers and physicians are “dual-agents”: they face dilemmas between their commitment to patients’ clinical needs and hospitals’ financial sustainability. We interviewed hospital managers and physicians in Israel and Germany. We were interested to find out if and how hospital professionals manage to balance and reconcile such dilemmas. The interviews showed that there are situations in which clinical and economic considerations are aligned; eg, when hospital payment incentivizes proper treatment. When considerations are misaligned, hospital professionals have identified a range of strategies to reconcile competing considerations. For example, sometimes efficiency can be improved, eg, by using cheaper, same quality, materials. Another strategy is the planning of the treatment course in advance, including an organized pre-operative phase to avoid last-minute cancellations and start surgery on time. Yet, if these strategies are used beyond a certain limit, the balance between considerations is disturbed, potentially undermining quality of care and creating unnecessary expenditures.

“I basically live in an ethical dilemma all the time. And this ethical dilemma is very simple. On the one hand, I want to give patients the best medicine here. On the other hand, I also need to keep this place financially balanced” (CEO, Israel).

###  Multiple Objectives in Hospitals 

 Complex institutions and organizations have various objectives, which are not always aligned.^1^ For example, financial sustainability and clinical quality/safety are two equally critical objectives. Failure in one of them may threaten the survival of the organization.^2^ The two objectives are often conflictive, but sometimes they can mutually reinforce each other.^3^ Different units, professional groups and individuals prioritize different objectives in an organization.^4,5^ For example, in healthcare organizations managerial staff may focus on economic goals whereas clinical staff may emphasize clinical safety. This can create dilemmas for staff when deciding which objective should prevail. According to the behavioral theory of the firm, there will always be latent and unresolved conflicts among the various objectives and players in organizations.^4^ Yet, hospital professionals must function amidst latent conflicts. While it has been understood that organizations have multiple objectives,^5^ these are often considered a) mutually exclusive; ie, independent from one another, and b) sequential; ie, one objective prevails and the secondary objective is attained only after the primary one.^2,3^

 In the hospital context, financial sustainability and quality/safety of care are two vital objectives. Hospital professionals commonly have specific objectives; eg, physicians cure patients, nurses care for them and managers control the hospital functioning and finances. Yet, at the same time, physicians can be deeply involved with the hospital’s managerial aspects, and managers may aim at high quality of care.^6^ When hospital professionals value multiple objectives as equally important, they may face dilemmas in their decision-making. Behavioral theory has so far overlooked situations in which objectives are interrelated and have similar priority.^2^ This study fills this gap by exploring whether financial sustainability and clinical quality/safety in hospitals are indeed conflicting objectives, and by learning about whether and how professionals pursue both.

###  Physician Agency and Dilemmas Between Economic and Clinical Considerations 

 Individual agency commonly denotes the capacity of individuals or groups to make their own choices and act independently. Proxy agency is defined here as a situation where a principal contracts with an agent over a desired outcome that is not directly observable by the principal.^7^ A principal-agent relationship occurs when one individual (the principal) engages another (the agent), and delegates decision-making power to the agent to perform a service on her behalf.^8^ Usually, the agent has more knowledge in the specific context, which creates problems of asymmetry of information. In the case of healthcare, the patient (principal) delegates decision-making power to the agents (the healthcare providers), assuming that the agents will use their knowledge to improve the health of the patient.^9-11^ Healthcare providers such as hospitals or physicians are perfect agents if they are fully committed to the patients. However, reality is more complex, and healthcare providers have additional commitments and considerations. For example, physicians consider their own professional or financial well-being, as well as their organizations’ objectives of financial sustainability.^12-17^ Hospitals too, may consider prestige, financial sustainability or research excellency as objectives.

 Payment mechanisms create economic incentives that may also influence healthcare providers’ treatment decisions regarding patients’ admission and treatment.^18-20^ In general, prospective/bundled payments such as budgets or salaries create incentives for providers to contain costs, deliver care efficiently, but also to limit the amount of resources used per patient or negatively select high-risk patients, who tend to use more resources.^21^ In retrospective/unbundled payments, in turn, the provider has no incentives to select (profitable) patients because each unit of care provided is fully reimbursed. However, there are no incentives to contain costs.^22^ Retrospective payments may thus encourage supplier-induced-demand; eg, providers suggesting unnecessary treatments.^23^ With perverse incentives potentially leading to service distortion, policy-makers attempt to balance incentives by mixing different types of payments,^7,24^ or by creating risk-adjusted payments such as capitations or diagnosis-related groups (DRGs).^22^ While the debate over how economic incentives are translated into healthcare providers’ actions is not new,^25-28^ it is important to note that providers are motivated by other considerations apart from financial ones, such as professional norms and status.^7^ Professional norms include, inter alia, quality of care and the regard for the patient.^29^

 Hospitals are organizations composed by their professionals, ie, doctors, nurses, chief physicians and management staff. Professionals play a central role in shaping organizational structures and behavior.^30^ Hospital professionals are “dual agents” when they are committed both to the patients and to the hospitals where they are employed.^31-33^ As “dual agents,” managers and physicians attempt to reconcile patients’ clinical needs and quality/safety of care with economic considerations to reach financial sustainability.^34^ When clinical and economic considerations do not align, “dual agents” may face dilemmas in decision-making regarding admission and treatment of patients. Should they be perfect agents to the patient or to the hospital? Should the treatment take into consideration the costs for the hospital? If dual agency and its dilemmas are not well coped with, they can lead to “moral distress,”^13^ professional burnout, inappropriate care or excessive spending.

 It is important to understand how hospital professionals, both managers and physicians, balance or reconcile economic and clinical considerations. Our study therefore aimed to address the following questions: (1) in which situations are economic and clinical considerations aligned and in which situations do dilemmas exist between economic and clinical considerations? (2) what strategies do hospital professionals use to balance these considerations in their daily decision-making? We assumed that economic and clinical considerations are conflictive but equally important objectives of hospitals.^2,4^ We further assumed that, as a result, hospital professionals are “dual agents” who frequently face dilemmas between economic and clinical considerations.^33,35^

## Methods

###  Research Setting: Why Germany and Israel?

####  Health Systems Similarities and Differences

 In this study we use qualitative data from interviews with hospital professionals in Germany and Israel. We chose these two countries because, on the one hand, they share some characteristics of healthcare systems: both have a mandatory, statutory health insurance (SHI) system, meaning that public funds are collected through mandatory earmarked contributions. These contributions are pooled and redistributed to competing non-profit insurers (called sickness funds or health plans), based on a risk-adjusted capitation formula. Insurers purchase hospital services in a setting of managed competition; ie, prices are regulated by the government, as well as the SHI basket of services and eligibility criteria.^36^ On the other hand, Germany and Israel differ substantially in their demographic composition, and in public funding and resources available to the health system (see Table S1 in Supplementary file 1). Compared to Israel, the German population is larger and its median age is higher. The German healthcare system relies more strongly on public funds and more resources are available, particularly in the hospital sector. Israeli hospitals work with fewer resources, lower rates of beds and nurses per population, high rates of bed occupancy, and shorter average length of stays (ALoS) compared to Germany.^37^ Basic population health indicators like life expectancy are similar in both contexts; average subjective health status among the German population is lower compared to the Israeli average.

####  Payment to Hospitals, Economic Incentives, and Reconciling Strategies

 Hospitals in both countries are paid based on activity. Germany implemented DRGs in 2003. Patients are classified according to DRGs and all admissions are paid by the same method.^38^ By way of comparison, Israel adopted procedure-related groups (PRGs) in the 1990s for certain procedures, and expanded this payment method between 2010-2015. In Israel patients are classified into a PRG according to the main procedure performed and paid on that basis; admissions without PRG-codes are paid based on per-diem.^39^ The shift from per diems and budgets to activity-based payments in both countries was meant to achieve a more appropriate and fair allocation of resources and increase transparency of measurement of activity.^40^ In Germany, an additional aim was to increase efficiency, given that ALoS was long and hospital capacities were not fully used. In Israel, efficiency was not a major aim, as hospitals’ resources were already stretched and they worked under pressure.^39^

 DRGs and PRGs create similar economic incentives for hospitals to: (*a*) increase the number of cases; (*b*) increase the income per patient; eg, by raising the number of income-generating procedures; and (*c*) reduce costs per patient; eg, by reducing the number of services provided per case, reducing the ALoS, or selecting low-risk patients. According to the literature, it is not clear how activity-based payments affect quality of care.^40-42^ For each economic incentive created, there may be positive and negative consequences. For example, increasing the number of cases can be positive, if treatment is clinically appropriate, or if it reduces waiting times. However, it can be negative if it leads to the performance of unnecessary or inappropriate procedures. Context plays a strong role in shaping responses to and outcomes of economic incentives and their relationship with clinical considerations. For example, whether hospitals have enough resources to “overprovide” care, or if waiting times are a concern and can be addressed by higher productivity.

####  Added Value of Comparative Design 

 Our study compares Germany’s ‘generous’ healthcare system with hospitals that have extra capacity, to the Israeli healthcare system, which is characterized by lower availability of public funds and stretched hospital resources. This comparative design allows us to test, first, whether economic and clinical considerations are inherently conflictive, or if contextual factors such as lower levels of resources play a role. If dilemmas between the two considerations are equally intense in both countries, this indicates that economic considerations are misaligned with clinical considerations across different contexts. Second, the comparative analysis allows examining the aforementioned structural similarities and differences, while being sensitive to context-specific phenomena such as treatment plans, patient case-mix and physicians’ preferences. We can thus better understand how structural patterns and particularities shape the dilemmas between different considerations; and we can recognize which strategies to manage the dilemmas between economic and clinical considerations are context-specific, and which ones may be ‘universal’ in that they are applied in different settings. Comparing two countries thus increases the generalizability of our results.^43^

###  Study Design and Participants

 This qualitative study is part of a broader study that assesses the impacts of PRG-based payments in Israel.^29,37^ We selected study participants from five hospitals each in Germany and in Israel respectively. Hospitals were sampled to provide maximum variation according to hospital characteristics that may influence the types of dilemmas that emerge, and reconciliation strategies developed. These characteristics are (1) type of ownership: public/private for-profit/private non-profit/health plan; (2) location: urban/center of the country vs. rural/periphery; and (3) size of hospital: big (more than 500 beds)/small (else).

 In order to supply rich and varied information, we selected respondents holding different professional roles in each hospital. When analyzing dual agency at the hospital level, we could not ignore the important role of physicians and managers in decision-making, particularly policies of admission and treatment. We included Chief Executive Officers (CEOs), Chief Financial Officers (CFOs), clinical and administrative managers, medical directors, ward directors (also called chief physicians) and practicing physicians who worked in inpatient surgical wards. We also chose to include CEOs and CFOs in order to test whether hospital managers are also “dual agents,” based on Minogue’s suggestion for future research.^34^ We limited our selection to procedural wards to allow better comparability of context and economic environment between the countries. In this paper, we refer to all positions except practicing physicians as “hospital managers.” We chose not to interview nurses and other hospital staff as they are less involved in decision-making related to admission and treatment policies.

###  Data Collection

 Data were collected through standardized open-ended in-depth interviews by RW, ED, DG, JK and MK. We built the interview protocol based on our research questions and the related literature. It was initially developed for the Israeli context and subsequently adapted to the German context. We further piloted and changed the protocols according to the interviewees’ reactions. The protocol included the following main topics: How do you take PRGs/DRGs into consideration in your daily work? What are the main considerations when you make your decisions? In which situations conflicts emerge between economic and clinical considerations? How do you cope with such conflicts? (See Supplementary file 1 for the last version of the interview protocols).

 We conducted face-to-face interviews with 46 hospital employees, 33 from Israel and 13 from Germany. Interviews in Israel were carried out between December 2017 and August 2018, and in Germany between March and August 2019. We invited 53 hospital employees from Israel and 50 from Germany to participate in our study via email and by phone, of whom 20 and 37 refused or did not respond, respectively. Our study participants included 5 hospital managers (CEOs), 8 CFOs, 5 clinical managers, 14 chief physicians/ward directors, and 14 physicians. It is important to note that CEOs in Israeli hospitals are physicians themselves, albeit without clinical responsibilities; whereas in Germany CEOs usually have no medical background. Ward directors in both countries act both as managers and practice medicine. Interviewees varied in ethnicity, age and seniority. Only four interviewees were female: one was a CFO, and three were physicians. Participants’ characteristics are presented in Table.

**Table:** Participant’s Main Characteristics

**Number of Participants**	**Israel**	**Germany**	**Total**
**Hospital Characteristics**
Hospital location			
Periphery/rural	18	8	26
Center/urban	15	5	20
Hospital size			
Big (≥500 beds)	29	5	34
Small (<500 beds)	4	8	12
Hospital ownership			
Public	8	6	14
Health plan	14	0	14
Non-governmental organization	11	6	17
Private for profit	0	1	1
**Interviewee Characteristics**			
Age, mean (range)	53 (39-67)	48 (27-72)	50.5
Gender			
Male	32	10	42
Female	1	3	4
Years in practice, mean (range)	10 (0.5-27)	3 (0.5-11)	6.5
Role			
CEO	4	1	5
CFO	6	2	8
Clinical manager/medical director	0	5	5
Chief physician/ward director	11	3	14
Physician	12	2	14
Ward/specialization			
Orthopedics	12	3	15
General surgery	5	3	8
Cardiovascular surgery	2	0	2
Ophthalmology	2	0	2
Urology	2	1	3

Abbreviations: CEO, Chief Executive Officer; CFO, Chief Financial Officer.

 Interviews were conducted in the local language (Hebrew and German respectively). They took place in the participants’ offices, lasted between 30 to 60 minutes, and were all audio-recorded and transcribed. All interviewees signed an informed consent form before the interview and were assured full confidentiality and anonymization.

###  Data Analysis

 We used thematic analysis, a suitable method to conduct applied research, as it identifies patterns of meaning across qualitative data in order to answer a predefined research question.^44,45^ It involved data reading, re-reading for familiarization, coding, building categories and themes, and revising them in an iterative process. The analysis approach was both deductive and inductive. Some codes and categories were initially built from the research questions, based on economic and organizational theory (theme 1 and related categories). Yet, the coding scheme was expanded and refined based on participants’ narratives (themes 2 and 3). To increase intercoder reliability, RW and NG read and coded all interviews in parallel, continuously developing the coding scheme both independently and jointly. DG, WQ and RB cross-validated and reconciled the coding. The analysis was done using “Atlas.ti 8” software. The original quotes in Hebrew and German were translated to English and their accuracy was validated by RW, DG, NG, WQ, JK and MK.

## Results

 We assumed that hospital professionals frequently face tensions between economic and clinical considerations. Indeed, we found that professionals face dilemmas, eg, *“when [the management] say[s]: ‘Listen, you use expensive equipment and… exceed our ability to balance the budget.’ I do not have a good way to deal [with this dilemma]*,* because I am committed to my employer, to the organization where I work, but I am also committed to patients” *(Physician in orthopedic ward, Israel). However, an important finding is that there are also situations where economic and clinical objectives are aligned, and financial sustainability and quality support each other. For instance, a clinically better treatment may also be cost-effective if it results in fewer complications and readmissions, and shortens ALoS: *“All the beauty [in DRG-based payments] is that it is a tool to promote excellency: the better you are, the faster you can discharge the patient, the more money you make. If your patient gets in trouble and gets infected*,* then there is a problem” *(CEO, Israel). In these cases, staff can be committed to both considerations simultaneously.

 This study focuses on the ways in which hospital professionals navigate economic and clinical considerations and mitigate dilemmas when they occur. In this regard, our analysis identified three main themes (see Figure). The first theme includes situations, in which dilemmas can be resolved and clinical and economic considerations are aligned. In this case, increasing efficiency is a way to address both considerations simultaneously. Increasing efficiency means actively changing treatment processes and technologies in order to reduce costs per patient, without hampering quality of care, or to improve quality of care without incurring additional costs. The second theme relates to strategies that mitigate dilemmas through changes in management. Reshaping management strategies denote the reorganization of treatment paths and coding without changing the treatment itself. The third theme subsumes professionals’ strategies to cope with unresolvable dilemmas by reframing decision-making such as focusing on different elements of ward or relying on interdisciplinary teams.

**Figure:**
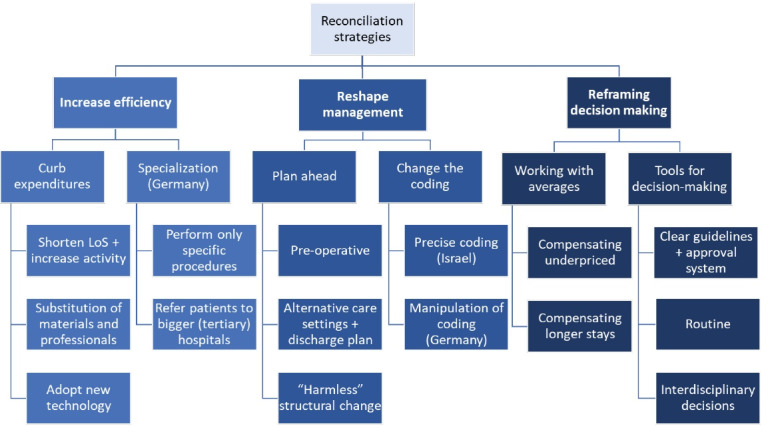
Theme and Categories of Reconciliation Strategies. Abbreviation: LoS, length of stay.

###  Theme 1: Increase efficiency 

 Efficiency can be improved when economic and clinical considerations can be truly reconciled in a win-win situation. We found two main strategies to improve efficiency: curbing of expenditures and specialization.

####  Curb Expenditures

 Participants in Israel and Germany reported that they can apply various measures to curb expenditures without hampering the quality and safety of care: shortening ALoS, increasing activity with the existing resources, substituting resources for lower-cost without undermining quality of care, and adopting technology that reduces costs.

####  Shorten Length of Stay and Increase Activity

 DRGs incentivize the shortening of length of stay (LoS). While reducing costs and allowing for a higher turnover of patients, shorter LoS can also be safer for patients as they are less exposed to hospital infections. Interviewees described that before the implementation of DRGs “*[ i ]t was a misery, both from an economic and a medical point of view. Patients stayed unnecessarily long in the hospital. And it isn’t desirable to spend your time in hospital, [in terms of] microbial load*,*etc*” (CEO, Germany).

 The shortening of LoS is attained by treating patients faster, reducing breaks and time between procedures, optimizing the use of operating rooms (ORs), paying less attention to details and spending less time with each patient. It enables faster treatment and recovery which is also desirable for the patients. However, it requires greater coordination of care with various levels and professionals: *“[T]o get the patient off the ventilators as quickly as possible, you need to start thinking about how to stop the anesthesia faster and detach the patient from the ventilator faster*. *This is an integrative action between the anesthesia department, the nurses and the doctors”* (Director of cardiovascular ward, Israel).

 However, the balance between curbing expenditures and quality of care is a delicate one: shortening LoS beyond a certain limit or working too fast can lead to poorer quality of care and safety faults such as complications and readmissions, which in turn have also negative economic consequences.

 Reducing LoS enables increasing the number of patients. Depending on the context, this can be a win-win situation where economic and clinical considerations are aligned. When many patients wait to be treated, a higher number of treated patients is better for all (under the assumption that procedures are performed in appropriate quality and they are appropriately reimbursed for). In Israel, one interviewee noted: “*I look at how to fill the slot [in the OR] in the right way that won’t waste time in an OR because… the more DRG [paid procedures] I make*,* the better for a hospital, right? But I also know that… there are loads of patients waiting [for a procedure]. So, the more I operate, the better… for the general public”* (Physician in orthopedic ward, Israel).

 Yet, here, too, there are two sides to the coin as increases in activity can lead to overtreatment: *“When the economist has already visited you three times and tells you*:* This year the numbers are (vehement knocking on the table) 5% below last year – then you start and grab anything that you can somehow fit into your discipline (laughs)”* (Clinical director, Germany). The concern of encouraging overtreatment is a particular concern in Germany, where waiting times are less of a problem.

####  Substitution of Materials and Professionals 

 Respondents from both countries reported substitution of expensive materials for cheaper alternatives and shifting simple tasks to non-skilled professionals as measures that lower the costs per case while maintaining quality of care. Substitution was usually negotiated between the management and end users (surgeons), to ensure the adequacy of alternative products: *“There is a particular implant that we use, and the [medical] center was able to get a very good business offer from a competing company. It is not that it is a defective implant or a less good implant*.* I said: ‘Wait, we have to support [the management] because it’s a very logical requirement. We’re not hurting our patients here, they’re getting surgery just the same.’ There is a problem that the new implant requires a little more ability from the surgeon. So, I said: ‘No problem, my compromise here is that this implant will only be used by surgeons who…feel safe to use because they have the surgical ability to work with it*’*” *(Director of orthopedic ward, Israel). However, the line between efficiency and inefficiency can be fuzzy and sometimes preferring cheaper materials *“is all well and good when that implant is cheaper than the other [with same quality]*.* But then the rate of complications [is higher and] the surgery takes longer*”(CEO, Germany). In such a case, the use of cheaper material is clinically and eventually also economically harmful.

 A creative strategy to improve efficiency reported by German professionals was substituting skilled professionals with unskilled workers for non-clinical tasks such as administrative tasks: *“We decided some time ago that we want to establish a project ‘Service staff on the ward.’ Nursing staff are an expensive resource. And there are also activities that the nursing staff does not necessarily have to provide*.* So, where we do not need care, we have service staff” *(CEO, Germany). An optimal allocation of resources, either workforce or materials, is a good way of reducing costs while maintain quality of care.

####  Adopt Innovative Technology

 Adopting new technology, albeit costly in the short run, may also improve efficiency as it can improve the quality of care and save costs in the long run. For example, a new laparoscopic technique may be costlier than open surgery, but it *“can shorten the length of the hospitalization, prevent complications, shorten the time of the surgery*,* and free the operating room faster”* (Physician in surgery ward, Israel).

 Here too, the balance between economic and clinical considerations is fragile, and when the costs of a new technology are not reflected in the DRG system, this may create barriers for its adoption. *“Green Laser, for example*,* is not used for prostate treatment in Israel. I promise you that if prostate treatment with Green Laser had a PRG tariff…*,* like mushrooms after rain, it would become a favorite operation and you would see everyone performing it”* (Director of urology ward, Israel).

####  Specialization

 In Germany, a central strategy to increase efficiency was specialization. Focusing on certain clinical fields allows professionals to become highly skilled and experienced in a limited portfolio of procedures, rather than spreading out on many different activities. Specialized professionals can work at high levels of quality and efficiency. One way of specializing was performing only specific procedures: *“We work like on a production line in high speed*. *We must always deliver the same quality […]. You must do the same thing in high quantities, as focused as possible – even if it’s boring – then you are good and then you have an advantage*”(Director of orthopedic ward, Germany).

 Another aspect of specializing in certain elective clinical fields is referring patients that do not “fit” the hospital portfolio to bigger (tertiary) hospitals. *“[If] a case is too complex for us, as long as it is not an emergency, I can say: ‘We are a hospital for basic and regular care*.* I recommend you […] a colleague who is more experienced or we refer you to a more specialized clinic. Please have the intervention done there.’ This way I do not harm the patient and have at the same time accounted for economic considerations”* (Chief physician at general surgery ward, Germany). It was important to the interviewees to point out that the patients get higher quality care elsewhere; ie, that this practice can effectively reconcile medical and economic considerations without harming patients. Yet, this equilibrium is also fragile, as specialization can be used as a tool for selecting attractive (overpriced) procedures or low-risk patients. If complex cases or underpriced procedures are systematically referred to bigger hospitals, these patients may face access barriers or longer waiting times.

###  Theme 2: Reshape Management

 Respondents reported that through reshaping management of wards and coding they could maximize revenues per case without changing treatment itself, thus mitigating dilemmas between economic and clinical considerations.

####  Plan Ahead

 A hospital is better able to provide appropriate care while reducing costs, the more it plans the treatment course of a patient in advance, and allocates resources and workforce strategically. For example, some hospital professionals pay attention to an organized pre-operative phase of the treatment to avoid last-minute cancellations and lost slots, and to start surgery on time. Others have well-planned discharge tracks that allow a smooth and fast discharge of patients, vacating beds more easily. *“We have preoperative [outpatient] clinics that bring patients in at least two weeks before the day [of the operation], to prepare the patient and avoid having last-minute problems. We try to plan ahead as many events as possible (…) If I detect problems two weeks in advance*,* I increase the output of the OR because I have less last-minute cancellations” *(Physician at orthopedic ward, Israel).

 Good post-operative care of patients moves those who are suitable in a timely fashion to alternative care settings such as recovery centers, rehabilitation, extended care facilities or home care. While vacating a hospital bed, those patients continue to receive nursing care, and another institution bears the costs of the continued long-term care instead of the hospital. *“On the fifth day after surgery, ‘whoop*,*’ the patient goes to a recovery or rehabilitation center or something, where there are less workers [than in a hospital] and they provide maintenance care, but he [the patient] was already discharged [from the hospital]” *(Director at cardiovascular ward, Israel).

 In both contexts, “harmless” structural changes of treatment paths allow increasing revenues without changing treatment itself.For example, in Israel, “*there are some manipulations of the system that he [the patient] can be transferred from the urgent system to an elective one”* (Physician at urology ward, Israel). For instance, treatments paid on PRG basis are shifted to outpatient/same day surgery in order to save ‘hotel’ costs, as the tariff is the same regardless of the setting of treatment. Similarly, patients who come to the emergency room but do not need urgent care are sent back home to perform diagnostic imaging at the health plans’ clinics and asked to return for elective surgery another time.

####  Change the Coding

 Changing the coding of activities in order to increase revenue per case was frequently reported. While interviewees in Israel reported efforts to improve the precision of coding of activities, interviewees in Germany often talked about how to manipulate coding. In Israel, most physicians were not aware of the potential of coding to improve the billing of activities:

*“To get the money you have to know how to code the procedure…. There are combinations of ICD-9 codes that are mandated by the MoH [Ministry of Health] and I figured out that physicians here really do not know this [MoH] directive so much*.* In fact, the hospital loses money because they don’t know how to make the right combination of codes. (…) what happens here today is like you have a supermarket full of products worth a lot of millions of shekels and you hire a cashier who does not know how to properly charge what people buy from the supermarket*.* You lose in the end”* (Director of orthopedic ward, Israel).

 In Germany, the DRG-based payment system is the main source of hospital income, and coding, supervision of coding, and reporting of coding plays a central role in many hospital professionals’ daily activities. *“[The colleague from controlling] takes a look: Hmmm*,* could you maybe change the order of the diagnoses? When you shift a diagnosis further up or to the second or third position, then something shifts in the DRG reimbursement*” (CEO, Germany). Considerable resources are dedicated to inspection and control of coding by hospitals, insurers and external institutions. There are internal supervisors who check the coding and look for items that can be further added to the coding in order to increase the revenue of the case. In addition, there are external examinations to alert against gaming or upcoding.

###  Theme 3: Reframing Decision-Making

 The third theme relates to re-framing as a strategy to reduce dilemmas between economic and clinical considerations. Re-framing implies a change of perspective rather than changes in clinical or managerial practices. For example, to shift the focus of decision-making or provide tools to improve decision-making.

####  Shifting the Focus: Working With Averages

 Hospital professionals in both countries shifted the focus of decision-making from the individual patient to a group of patients within one DRG in order to ‘work with averages.’ This way, they could justify financially unattractive activities as they were eventually balanced out with many attractive ones. For example, professionals treated many low-risk or overpaid cases in order to create ‘reserves,’ which then allowed them to treat a complex patient or to perform underpaid procedures: *“It is always a mixture of large surgeries*,* which possibly have a large yield, and many small things, with which you can do a lot of good, [but] where you possibly end up losing out” *(Chief physician of orthopedic ward, Germany).

 Hospital professionals thus accommodate economic and clinical considerations of an entire ward or a certain period of time, instead of trying to solve the dilemma for each patient. Another strategy was looking at a DRG as a bulk of cases. One interviewee described that “*there can be one [patient] who stays [hospitalized] for a long time and [another] one who stays a very short time*.* But the average tends to the optimal LoS for a particular procedure*” (CFO, Israel). This framing justifies treating each patient according to his/her particular clinical needs, as long as the ward meets the overall average target.

####  Tools for Decision-Making 

 Dilemmas between different considerations can be mitigated with clear treatment guidelines, instructions and information, that constitute a tool-kit for decision-making. This tool-kit may provide criteria for when to use costly materials or procedures, when to prioritize clinical considerations or when economic considerations should prevail. It can help managers and physicians to make decisions by providing them with evidence and agreed-on criteria, thus relieving them from having to weigh the different considerations based only on their own values and knowledge: *“Once, every second child who fell would undergo a brain CT*.* Today, we just supervise [the child]. Now there are criteria [to treat falls] that have medically changed things. It [the new guidelines] is also more economic, because not everyone needs a CT”* (CEO, Israel).

 Instructions, clear processes of treatment and the need for the approval of the management can ease the daily decision-making for hospital professionals, because they can rely on organizational rules and decisions that were consolidated and agreed on by various players. Similarly, respondents reported that “routine” mitigated their dilemmas because decisions were made once and further replicated, exempting players from new decisions and new dilemmas related to each patient. The drawback of having rigid guidelines for treatment or a steady routine is that it can undermine the flexibility needed in treating certain patients according to their particularities, and reduce the autonomy of health professionals.

 Joint, multidisciplinary decision-making among professionals from various disciplines seems to be a strong reconciling strategy because it takes into consideration various points of view, with different priorities, thus leading to more robust decisions:“*Now, we don’t allow any more that only a surgeon decides on the basis of the X-ray image…. Instead, we hold so-called indication conferences, not only the surgeons sit at the table, but also the neurologist*,* the physiotherapist, the pain therapist and even the psychosomatics” *(Clinical director, Germany) Moreover, instead of a single professional coping with the dilemma, this burden is thus shared among the group.

## Discussion

 This study fills a gap in the knowledge on HOW hospital professionals, ie, managers, CFOs, chief physicians and practicing physicians, mitigate dilemmas by balancing or reconciling economic and clinical considerations in the context of activity-based payment. Focusing on Israel and Germany as case studies, we unpack three types of strategies: (1) reconciliation between economic and clinical considerations through increasing efficiency, which is possible only in those situations when there is no inherent conflict between these objectives. This is the case when activity-based payment incentivizes proper treatment; (2) the mitigation of dilemmas by reshaping managerial practices, such as treatment paths and coding; and (3) balancing considerations through reframing the focus of decision-making to bigger units of analysis. Similar to hospitals, many types of organizations face structural dilemmas, particularly between the need for high-quality activities and safeguarding resources. Maintaining the balance between opposing considerations is not only important to help professionals make decisions, but is also key for organizational resilience.^46^ Our findings may provide some guidance for professionals on ways to mitigate dilemmas and for organizations to improve their resilience.

###  Touching on a Fragile Balance

 Our analysis indicates that in the context of DRGs and PRGs, the reconciliation between economic and clinical considerations can contribute to more efficient and appropriate care. However, our results also suggest that some reconciliation strategies touch on a fragile balance between high-quality care and financial sustainability. If overdone, they are liable to achieve the opposite of the intended objective. For example, ‘specialization’ and ‘working with averages’ can improve efficiency or allow hospitals to treat economically unattractive cases. But if taken too far, they can turn into selection of patients, with the risk of negative impacts on patients with higher risk or in need of underpaid procedures. Similarly, substituting materials, skilled professionals or shortening LoS are strategies that can potentially lead to poorer safety or quality of care, if they go beyond a certain limit. Decision-makers should carefully monitor the strength of payment incentives as they may push hospital professionals to go beyond the point at which clinical and economic considerations are aligned.

 Strategies included in the themes ‘reshaping management,’ and ‘reframing decision-making,’ are seen as more robust than strategies included in the theme ‘increasing efficiency.’ These do not involve changing the treatment or reordering the activities of a certain ward. Thus, the chances of negative unintended consequences are lower.^16,47^ Working in an (interdisciplinary) group where professionals consult with each other also reduces uncertainty and ambiguity in decision-making, mitigating dilemmas between economic and clinical considerations.^7^

###  Different Professional Roles and Different Considerations Can Align

 Our analysis shows that the professional role influences the choice of reconciling strategy. For example, physicians focused more on the clinical practice and how to solve dilemmas at the patient level (eg, reducing LoS), while chief physicians could take into consideration the entire ward and “work with averages,” and CEOs could rethink the coding policy. Yet, professionals in all roles developed strategies to mitigate dilemmas, including professionals who are not physicians such as CEOs without clinical background (in Germany) and CFOs.

 Hospital managers and physicians are often considered separate groups, with opposite values and objectives.^48,49^ According to this viewpoint, the two groups are in an inherent conflict, with managers seen as trying to manipulate physicians’ clinical decisions in order to meet economic objectives.^50^ Yet, sometimes physicians have also managerial tasks, and managers are also clinicians. In these cases, hospital professionals are not only “dual agents” committed to more than one principal, but also ‘professional hybrids.’ The concept of professional hybrids originated in the literature of sociology of professions, with the introduction of market-like mechanisms to public services^51^ and describe professionals that perform duties outside their profession, ie, managers who combine a professional background with managerial skills and responsibilities, or clinicians who are also managers or leaders.^1,52^ Generically, it refers to professionals occupying hybrid roles, with complex identities embedded in different professional groups^53^ or two institutional logics that develop and coexist in one organization.^51^ In the context of health providers, the concept of ‘professional hybrids’ emerged with the introduction of managerial tasks to hospital physicians when hospitals started bearing financial responsibility. It occurred in the United Kingdom upon the establishment of hospital trusts^54^ and in other countries upon the adoption of DRG-like payments^30^ in the 1990s, and then expanded to other contexts and settings such as primary care, mental health, and non-health contexts.^53,55-57^ Most of the literature explores how physicians adopt managerial roles.^51,54,55,58-60^

 This body of literature is helpful to understand our findings, because it explores the misalignment between management and medicine, and dilemmas that these professionals face due to their dual roles.^61-67^ We found that ward managers/chief physicians in both countries were the professionals who reported facing dilemmas most frequently. They are the typical ‘professional hybrids’ with dual roles of managers and clinicians, and mediate between the hospital management and physicians.^51,54,58^ Yet, our findings highlight that, while managers focus more on economic considerations than physicians, they also have clinical considerations in mind. Similarly, physicians do not ignore economic considerations. Our study thus deconstructs the dichotomy of two opposed professional groups with evidence about strategies used both by hospital managers and physicians to reconcile clinical and economic considerations when they misalign, highlighting how these professionals combine two institutional logics in hospitals. We extend the literature with evidence that not only physicians or medical directors are “professional hybrids,” but CEOs and CFOs may also be, when they adopt the clinical logic in their profession, by attempting to balance clinical considerations in their decision-making.

 Much literature on ‘professional hybrids’ relates to the dual logics of professionalism and managerialism and how professional hybrids prioritize or combine contradictory institutional logics through their identities, and subjective processes of sensemaking and interpretations.^1,67,68^ Our findings complement the existing knowledge by exploring the specific dilemma between economic and clinical considerations in hospitals. While professional hybrids may be perceived as more effective and credible due to their knowledge from both fields,^54,59^ some studies show that the ‘competing logics’ and role ambiguity are a hindrance, rather than a potential for reconciliation. For example, Andersson and Liff^64^ argue that reconciliation between managerial and clinical considerations led to poorer quality of psychiatric care in Sweden. Studies conclude that professional hybrids typically adopt one role over the other,^69^ and that has negative implications for the other professionals within the same organization, at least in healthcare organizations.^70^ Few studies to date explore HOW ‘professional hybrids’ reconcile economic and clinical considerations in their daily* practice*, beyond their internal logic or sensemaking.^51,71^ Our findings suggest that economic and clinical considerations are less dichotomous than hitherto presented in the literature.^1,34^ The dilemmas between the different considerations are, in fact, not necessarily negative if hospital professionals are able to realign them, eg, by improving planning, increasing efficiency or implementing tools for decision-making.

###  (Mis-)Alignment of Considerations and Reconciliation Strategies Depend on the Context

 The comparison between Germany and Israel revealed that economic and clinical considerations can align — and enable greater efficiency — both in countries with high and low resource hospital settings. Our study also shows that the context and work environment can play a key role in determining if considerations can be aligned or not. For example, DRGs provide incentives to increase the number of patients. In Germany, where hospital capacity is high, this can result in treatment distortion/overtreatment (misaligned considerations); whereas in Israel, where hospital resources are relatively scarce, treating more patients shortens waiting times and promotes timely care (aligned considerations).

 Comparing Germany to Israel also highlights the fundamentally similar dilemmas faced by hospital professionals, despite different levels of resources, as well as commonalities in reconciliation strategies. The comparison further allowed us to differentiate between the implications of the same reconciliation strategies in healthcare systems and hospitals with fewer or more resources. Some reconciliation strategies depended on the context to an extent that they were not applicable in both countries. For example, specialization in specific clinical fields was reported only by German participants in small hospitals. One possible explanation is related to the hospital market structure: in Germany there are many small hospitals that are independent non-profit or private for-profit. They can specialize because there is enough offer to meet the demand. In Israel, specialization is less feasible because there are few, big, public, and general hospitals. Also, moving patients to alternative care settings is more feasible in Germany; while in Israel rehabilitation settings are less available and create bottlenecks for discharging patients and shortening LoS.^29^ This may explain why Israeli respondents often reported on planning the pre-operative part of the treatment as a managerial reconciliation strategy; whereas their German counterparts rather focused on the post-operative part.

 The perpetually underfunded environment of Israeli hospitals may also explain why Israeli interviewees seemed less critical about “working faster” than their German counterparts. This may indicate that Israeli professionals are more used to work under pressure and resource constraints than their German colleagues. Wehkamp and Naegler^50^ support these findings, reporting that physicians in German hospitals complain about pressure, increased workload, acceleration, which led to stress and burnout. Regarding other hospital characteristics, we have not found big differences in responses between different types of ownership and location.

###  Limitations

 This study has two main limitations. First, we rarely heard about strategies that fail to reconcile between the two considerations. It is likely that responses were biased towards successful strategies. It is known from the literature that there are strategies that fail, and physicians may either make non-optimal clinical decisions, or make decisions that clash with their other commitments.^13,16^ Yet, our objective was to learn ‘what works,’ instead of ‘all strategies used.’ Second, while interviewees may have faced various types of dilemmas, we have chosen to focus on those caused by misalignment between economic and clinical considerations. Interviewees mentioned also dilemmas between social and clinical needs, managerial and clinical needs. However, these were beyond the scope of this paper.

## Conclusions and Practice Implications

 All payment mechanisms, including activity-based payments, can – but not always do – create situations, where health professionals face dilemmas between economic and clinical considerations. Clinical and economic considerations align when activity-based payment incentivizes proper treatment. Dilemmas in decision-making are not necessarily negative, if professionals manage to reconcile conflicting considerations and thus create a win-win situation. This is the case when efficiency can be improved, eg, by curbing unnecessary expenditures or specializing in certain procedures. When considerations misalign, hospital professionals apply strategies to balance between them such as ‘reshaping management’ and ‘reframing decision-making.’ However, health administrators and leaders should be cautious, as some reconciliation strategies are successful only up to a certain limit. Beyond this limit, they can lead to negative consequences: if economic considerations are overemphasized and clinical considerations ignored, these consequences can include the selection of patients, poorer quality of care or overtreatment. Disregard for economic considerations, on the other hand, may lead professionals to incur unnecessary costs and squander resources.

## Acknowledgements

 We thank Elad Daniels, Julia Kelek and Michelle Kutscher for helping collect the data and Yael Paldi for helping analyze the Israeli data. We thank all our interviewees for their time and attention.

## Ethical issues

 The research, methods and interview protocol were approved by the ethics committee of Ben-Gurion University of the Negev (approval no. 1580-1). All interviewees signed an informed consent form before the interview and were assured full confidentiality and anonymization.

## Competing interests

 Authors declare that they have no competing interests.

## Authors’ contributions

 RW acquired funding; RW and WQ conceived and designed the work; RW, NG, WQ, and DG analyzed the data and drafted the work. All authors revised the work critically for important intellectual content. All authors approved of the version to be published; and agree to be accountable for all aspects of the work in ensuring that questions related to the accuracy or integrity of any part of the work are appropriately investigated and resolved.

## Disclaimer

 The authors’ state that the views expressed in the submitted article are our own and not an official position of the institution or funder. This data was not published elsewhere. It was be presented as a poster at the 19th German congress of health services research and The Virtual 16th World Congress on public health in 2020.

## Funding

 This work was supported by the Israel National Institute for Health Policy Research (NIHP) [Grant number 77–16]. The main author thanks Minerva Stiftung for the support with the Minerva Fellowship. The NIHP and Minerva Stiftung had no involvement in the study design; in the collection, analysis and interpretation of data; in the writing of the report; or in the decision to submit the article for publication. The researchers are independent of the funders.

## Supplementary files

Supplementary file 1. Summary Profile of the Israeli and German Health Systems, and Interview Protocol.
