# Supplementary material for: Dual Agency in Hospitals: What Strategies Do Managers and Physicians Apply to Reconcile Dilemmas Between Clinical and Economic Considerations?
Source: Int J Health Policy Manag. 2021 Aug 15;11(9):1823–34. doi: 10.34172/ijhpm.2021.87 (PMC9808238; doi:10.34172/ijhpm.2021.87)
Supplement: Supplementary file 1 — Summary Profile of the Israeli and German Health Systems, and Interview Protocol. [file ijhpm-11-1823-s001.pdf]

Article title: Dual Agency in Hospitals: What Strategies Do Managers and Physicians Apply to Reconcile Dilemmas Between Clinical and Economic Considerations?

Journal name: International Journal of Health Policy and Management (IJHPM)

Authors' information: Ruth Waitzberg[1,2,3]*, Nora Gottlieb[3,4], Wilm Quentin[3,5], Reinhard Busse[3,5], Dan Greenberg[2]

[1]The Smokler Center for Health Policy Research, Myers-JDC-Brookdale Institute, Jerusalem, Israel.

[2]Department of Health Policy and Management, School of Public Health, Faculty of Health Sciences, Ben-Gurion University of the Negev, Beer-Sheva, Israel.

[3]Department of Health Care Management, Faculty of Economics & Management, Technical University Berlin, Berlin, Germany.

[4]Department of Population Medicine and Health Services Research, School of Public Health, Bielefeld University, Bielefeld, Germany.

[5]European Observatory on Health Systems and Policies, Brussels, Belgium.

(*Corresponding author: ruthw@jdc.org)

Supplementary file 1. Summary Profile of the Israeli and German Health Systems, and Interview Protocol.

1.1 *Table 1: Overview of the main characteristics of the German and Israeli healthcare system and hospital sector, 2017*

	Indicator*	Germany	Israel
Population	Population	83 million	8 million
	Share of aged 65+	21.5%	11.5%
System characteristics	Total expenditure on health as % of GDP	11.2	7.4
	Expenditure per capita (US$ PPP)	5,848	2,666
	Public share of THE	84%	64%
	Total expenditure on health by sources of funding	Statutory health insurance on 15% of income (57%); other social insurances (17%); government funding from general taxes (4%); private funding (22%)	Statutory health insurance on 5% of income (24%); government funding from general taxes

			(40%); private funding (36%)
	Payer of health services	105 non-profit competing health plans	4 non-profit competing health plans
Hospital resources	Number of general hospitals	1,592	35
	Rate of acute and rehab care beds per 1,000 population	6.1	2.2
	Hospital beds in for-profit private hospitals, per 1,000 population	2.4	0.4
	Professional Nurses and Midwives in hospitals**, Density per 1,000 population (FTE)	4.23	2.83
	Physicians in hospitals**, Density per 1,000 population (FTE)	2.03	2.08
	Bed occupancy rate (acute care)	80	94
	Average lengths of stay (acute care, days)	7.6	4.0
	Hospital discharge rates, per 100,000 population	25,478	15,636
Health status	Life expectancy at birth (years)	81.1 (83.4 females; 78.7 males)	82.6 (84.6 females; 80.6 males)
	Infant mortality per 1,000 live births	3.3	3.1
	Perceived health status good, very good (% aged 15+)	65	74

(*) all data refers to year 2017 unless otherwise stated; (**) Data refer to practicing physicians in 2017. Practicing physicians are defined as those providing care directly to patients. FTE = full time equivalent. PD = per diem; DRG = Diagnostic-related groups; PRG = Procedure-related groups.

Sources:

Blumel M, Spranger A, Achstetter K, Maresso A, Busse R. “Germany: Health system review”. *Health Systems in Transition*, 2020; 22(6). https://eurohealthobservatory.who.int/publications/i/germany-health-system-review-2020 , Accessed August 4, 2021.

OECD statistics, 2020 https://stats.oecd.org/ Accessed August 4, 2021.

OECD, “Health at a Glance 2019: OECD Indicators,” OECD Publishing, Paris, Nov. 2019. doi: 10.1787/4dd50c09-en. Accessed August 4, 2021.

Rosen, B. Waitzberg, R. and Merkur, S. “Israel: Health System Review,” *Health systems in transition*, 2015; 17(6). https://eurohealthobservatory.who.int/countries/israel/ Accessed August 4, 2021.

The Federal Statistical Office: https://www.destatis.de/EN/Press/2019/03/PE19_109_23611.html .

1.2Interview protocol for Israel

General introduction

- Thank you for agreeing to be interviewed and contribute to our study on the matter …
- My name is …. And I work at ……
- This study is part of a doctorate in cooperation with Ben-Gurion University and the Technological University of Berlin.
- **The subject of the study** is the change in the method of payment to hospitals. Between 2010 and 2014, the Israeli Ministry of Health changed the method of payment to hospitals, from payment according to the duration of hospitalization to payment according to the procedure; this is a sort of package that includes all the costs of the procedure, regardless of the duration of the hospitalization.
- **The study goal** is to learn how the change in the method of payment affects the running of the hospital and in particular how the treatment staff views this change.
- During the interview, we will ask you to share your experiences and insights about the study goal and anything else that you think is relevant to the various aspects.
- We have received approval from the Ethics Committee to conduct the study. We are therefore required to ask your approval and signature on the informed consent form.
- We would also be grateful for your approval to record the interview, since we will not have time to record everything in writing.
- The interview will last about one hour
- We assure you of complete confidentiality regarding your identity at all stages of the study
- You are free to stop the interview at any stage you wish.

Questions for hospital directors

1. How old are you? How long have you held this position?
2. How has the change affected your role as director and the running of the hospital?
3. Following the change, what new messages have you sent to the heads of departments? To the management of the operating rooms? And to which departments?
4. By what means do you send the messages?
5. Is it difficult to pass on messages?
6. How did the relevant departments react to your instructions?
7. How do you supervise the departments?
8. How has the change affected negotiations between the health plans (the agreements between the health plans)?
9. Has the change affected your relationship with the heads of departments and the physicians?

Questions for department heads

1. How old are you? How long have you held this position?
2. How has the change affected your role as head of department and the running of the department?

3. Following the change, what new messages have you received from the hospital directors?
4. Following the change, what new messages have you sent to the physicians in your department?
5. By what means do you send the messages?
6. How did the physicians react to your instructions?
   Probe: Is it difficult to convey messages?
7. How has the change affected the way you work?
8. Has the change affected your relationship with the physicians?

Questions for physicians

1. How old are you? How long have you held this position?
2. Who decides who will be operated on? What procedures are performed and when are patients discharged?
3. Has what you described been the case since you started working here?
4. Has there been a change in prioritizing procedures in the department schedule?
   (Only if no answer: Do you have out-of-hour sessions? If so, has there been a change in the type of procedures performed out of hours?)
5. What change has there been in the extent of your autonomy? Type of considerations and decisions?
6. Are the outcomes of your work measured? What aspects are measured?
7. How is what you have told us until now, together with the change in the method of payment, reflected in messages received from management or in management's expectations?
   (Only if no answer: For example in recording procedures, clinical instructions, use of certain materials or equipment)
8. How is success measured? And how does the hospital or the head of department acknowledge your success?
9. *If there is still time: How is your relationship with the department management and hospital management?*

Questions for finance directors

1. How old are you? How long have you held this position?
2. How has the change affected your role as finance director and the running of the hospital?
3. Following the change, what new messages have you sent to the heads of departments? To the general director?
4. And to which departments?
5. By what means do you send your messages?
6. Is it difficult to convey messages?
7. How have the relevant departments reacted to your instructions?
8. How do you supervise the departments?
9. How has the change affected negotiations between the health plans (the agreements between the health plans)?

Dual agency among hospital workers

10. Has the change affected the relationship between management and the heads of departments and physicians?

1.3Interview protocol for Germany

General introduction

- Thank you for agreeing to be interviewed and contribute to our study on DRGs.
- My name is …. And I am student at ……
- This study is part of a bi-national doctorate in cooperation with Ben-Gurion University from Israel and TU Berlin.
- **The objective of the study** is hospital workers' perspectives about DRGs.
- During the interview, we will ask you to share your experiences and insights about the topic and anything else that you think is relevant to DRGs and how people work in this hospital.
- We have received approval from the Ethics Committee to conduct the study. We therefore ask your approval and signature on the informed consent form.
- We would also be grateful for your approval to record the interview, since we will not have time to write everything.
- The interview will last about one hour
- We assure you of complete confidentiality regarding your identity at all stages of the study, findings will be completely blinded and the report will have aggregated data and de-identified data.
- You are free to stop the interview at any stage you wish.

Questions for hospital directors/ CEO/ CFO/ Medical Directors

1. How old are you? How long have you held this position?
2. How do you take DRGs into consideration in your daily work?
   Probe: think about all the patient path in the hospital
3. How do ward directors/ chief physicians and physicians take DRGs into consideration in their daily work?
4. How do you communicate your decisions to hospital workers? What workers?
   Probe: How do your decisions reach hospital workers?
5. How do ward directors/chief physicians react to your decisions?
6. What are your main considerations when you make your decisions?
7. In what situations your considerations may not be aligned?
8. How do you take quality into consideration in your daily work?
9. Is there any other thing that you want to add? Is there anything that I didn't thought about and should have thought? Is there any question that I didn't ask, that I should have asked?

Questions for ward directors/ chief physicians

1. How old are you? How long have you held this position?
2. How do you take DRGs into consideration in your daily work?
   Probe: think about all the patient path in the hospital
3. How do physicians take DRGs into consideration in their daily work?
4. You are in between the management and the physicians, tell me how that works.

Probe: How do your superiors' communicate their decisions to you?
Probe: How do you communicate your decisions to physicians?
Probe: How do your decisions reach physicians?
Probe: How do you communicate your superiors' decisions to physicians?
Probe: How do your decisions reach physicians?
Probe: How do physicians react to your decisions?

5. What are your main considerations when you make your decisions?
6. In what situations your considerations may not be aligned?
7. How do you take quality into consideration in your daily work?
8. Is there any other thing that you want to add? Is there anything that I didn't thought about and should have thought? Is there any question that I didn't ask, that I should have asked?

Questions for physicians

1. How old are you? How long have you held this position?
2. How do you take DRGs into consideration in your daily work?
   Probe: think about all the patient path in the hospital
3. How do your superiors' communicate their decisions to you
4. How you react to their decisions?
5. What are your main considerations when you make your decisions?
6. In what situations your considerations may not be aligned?
7. How do you take quality into consideration in your daily work?
8. In what situations patients don’t agree with you / your decisions?
9. Is there any other thing that you want to add? Is there anything that I didn't thought about and should have thought? Is there any question that I didn't ask, that I should have asked?
